# Induction of Apoptosis by Sinulariolide from Soft Coral through Mitochondrial-Related and p38MAPK Pathways on Human Bladder Carcinoma Cells

**DOI:** 10.3390/md10122893

**Published:** 2012-12-18

**Authors:** Choo-Aun Neoh, Robert Y.-L. Wang, Zhong-Hao Din, Jui-Hsin Su, Yu-Kuei Chen, Feng-Jen Tsai, Shun-Hsiang Weng, Yu-Jen Wu

**Affiliations:** 1 Department of Research, Pingtung Christian Hospital, Pingtung 90059, Taiwan; E-Mail: neohca@gmail.com; 2 Department of Biomedical Sciences and Research Center for Emerging Viral Infections, Chang Gung University, Taoyuan 33302, Taiwan; E-Mail: yuwang@mail.cgu.edu.tw; 3 Graduate Institute of Applied Healthy and Biotechnology, Meiho University, Pingtung 91202, Taiwan; E-Mail: nmm10023@yahoo.com.tw; 4 National Museum of Marine Biology and Aquarium, Pingtung 94446, Taiwan; E-Mail: x2219@nmmba.gov.tw; 5 Department of Food Science and Nutrition, Meiho University, Pingtung 91202, Taiwan; E-Mail: x00008396@meiho.edu.tw; 6 Department of Beauty Science, Meiho University, Pingtung 91202, Taiwan; E-Mail: x00002036@meiho.edu.tw; 7 Department of Hospitality Management, Meiho University, Pingtung 91202, Taiwan; E-Mail: x00009520@meiho.edu.tw

**Keywords:** sinulariolide, bladder cancer cells, mitochondrial-related pathways

## Abstract

Sinulariolide, an isolated compound from the soft coral *Sinularia flexibilis*, possesses the anti-proliferative, anti-migratory and apoptosis-inducing activities against the TSGH bladder carcinoma cell. The anti-tumor effects of sinulariolide were determined by 3-(4,5-cimethylthiazol-2-yl)-2,5-diphenyl tetrazolium bromide assay, cell migration assay and flow cytometry, respectively. Sinulariolide inhibited the growth and migration of bladder carcinoma cells in a dose-dependent manner, as well as induced both early and late apoptosis as determined by the flow cytometer. Also, the sinulariolide-induced apoptosis is related to the mitochondrial-mediated apoptosis via caspase-dependent pathways, elucidated by the loss of mitochondrial membrane potential, release of cytochrome *C*, activation of caspase-3/-9, Bax and Bad, as well as suppression of Bcl-2/Bcl-xL/Mcl-1. Detection of the PARP-1 cleaved product suggested the partial involvement of caspase-independent pathways. Moreover, inhibition of p38MAPK activity leads to the rescue of the cell cytotoxicity of sinulariolide-treated TSGH cells, indicating that the p38MAPK pathway is also involved in the sinulariolide-induced cell apoptosis. Altogether, these results suggest that sinulariolide induces apoptosis against bladder cancer cells through mitochondrial-related and p38MAPK pathways.

## 1. Introduction

Urothelial carcinoma, a cancer derived from transitional epithelium, occurs mainly in the urinary bladder, ureters, or renal pelvis [1]. It is the fifth most common malignant neoplasm worldwide. It has the second highest mortality rate among urological neoplasms [2,3]. Clinical evidences show that bladder tumors account for 90%–95% of urothelial carcinomas. Approximately 90% of human bladder tumors are present as transitional cell tumors [4,5]. It has been reported that transitional cell tumors are among the most highly prevalent cancers diagnosed in adults, and are a common cause of death through genitourinary tumors [6,7]. Hence, it is believed that transitional cell carcinoma in the elderly population is becoming increasingly important. More important, it has been reported that the percentage of female patients with cancer rises with increasing age [8].

There are two ways that transitional cell carcinoma spreads into the nearby tissue [9]. The first way is usually found in the epithelial cells, which line the body cavity and many of the passageways that exit the body. The second way of spread is through the lymphatic systems. Although surgery is the most common means to treat transitional cell carcinoma in the bladder, other types of therapy called immunotherapy and chemotherapy are often used in treating transitional cell carcinoma. The intravesical bacillus Calmette-Gueérin (BCG) is among the most used agent. When BCG is applied in bladder tumors, the body begins an immune response that sometimes destroys the tumor [10,11]. Nowadays, the combination usage of chemotherapy and radiation is being considered as a therapy for the transitional cell carcinoma despite the fact that the method has not yet been studied [12,13]. Therefore, it is essential to find new diagnosis and treatment measures for superficial transitional cell carcinoma. 

The specific induction of cancer cell programmed death triggering by apoptosis using chemotherapy is highly studied and most critically benefits cancer therapy development [14]. Apoptotic processes can be triggered either via the plasma membrane (extrinsic pathways) or within cells (intrinsic pathways) [15]. The intrinsic signaling pathways that initiate apoptosis involve a large number of intracellular signals which are located in either the mitochondria or endoplasmic reticulum (ER) [16]. During apoptosis, pro-apoptotic members of the Bcl-2 family, like Bax, alternate mitochondrial membrane potential and release mitochondrial apoptotic factors [17]. Cytochrome *C* release from mitochondrial inter-membrane spaces then activates caspase-9, further activating the downstream effector caspase-3 in order to cleave poly (ADP-ribose) polymerase-1 (PARP-1) that is further activated [18]. In mammals, there are three major groups—p38MAPK, c-Jun NH2-terminal kinase (JNK), and extracellular signal regulated kinase (ERK)—identified within the mitogen-activated protein kinase (MAPK) signaling pathways [19]. MAPK signaling pathways regulate a variety of physiological processes, such as cell differentiation, cell growth and apoptotic cell death in response to a variety of stress-related stimuli [19,20]. 

**Figure 1:**
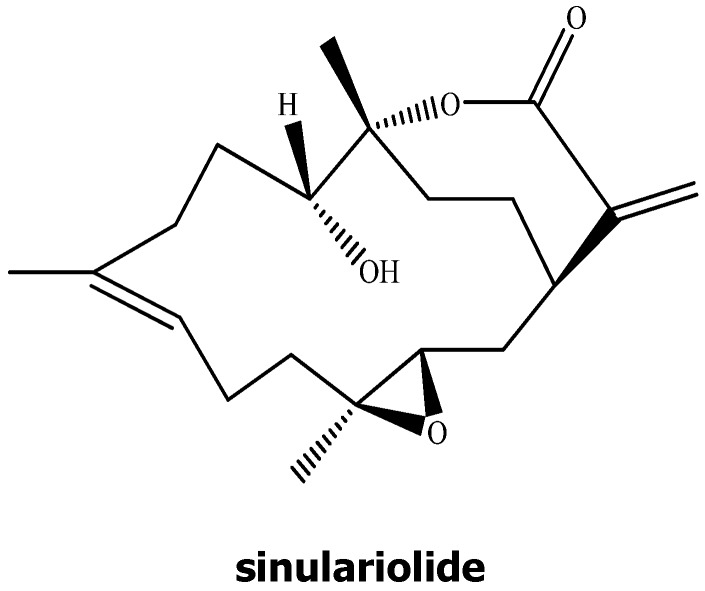
Chemical structure of sinulariolide.

The therapeutic applications of natural products isolated from marine soft corals have been widely investigated [21,22,23,24]. Several compounds such as diterpenes, diterpenoids, and prostanoids have been isolated from soft corals. Despite its unknown mechanism, these compounds have been reported to exhibit the anti-cancer effects through the induction of apoptosis and cytotoxic against different cancer cell lines, such as prostate, breast, colon, melanoma, liver, oral cancer and cervical cell lines [25,26,27,28,29,30]. Only a few reports addressed the activities of compounds from natural products on human bladder carcinoma cells. In this study, a cembrane-based diterpenoid, sinulariolide (Figure 1), was isolated from *Sinularia flexibilis* and subsequently examined for cytotoxic effects on the TSGH cells. This compound, sinulariolide, possesses anti-proliferative, anti-migratory as well as apoptosis-inducing activities against the TSGH bladder carcinoma cell. In addition, it was found that the apoptosis induced by sinulariolide is related to mitochondrial-mediated apoptosis via caspase-dependent pathways. These results provide valuable information for understanding the biochemical aspects of the cytotoxic effects of sinulariolide on TSGH cells and will help drug development and progression monitoring of human bladder carcinoma.

## 2. Results

### 2.1. The Anti-Proliferative and Anti-Migratory Effect of Sinulariolide on TSGH Cells

**Figure 2:**
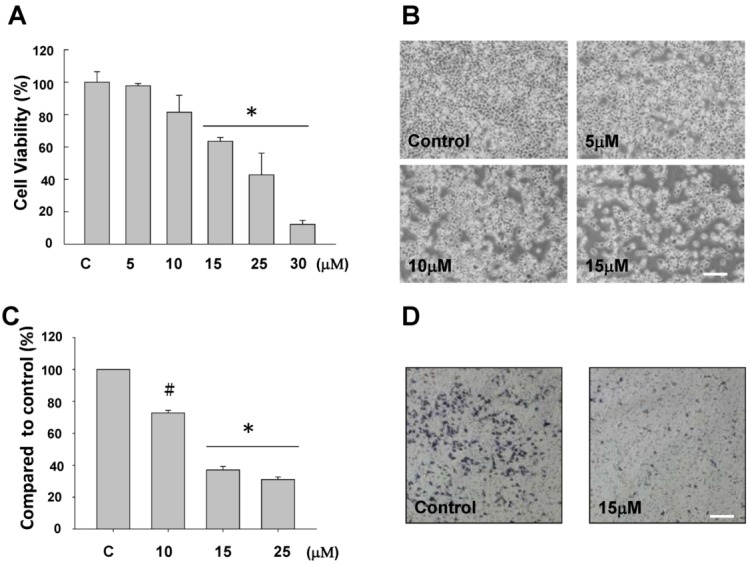
Evaluation of the anti-proliferative and anti-migratory effects of sinulariolide on TSGH cells (**A**) The viability of TSGH cells were dose dependently suppressed by treatment with 5–30 μM sinulariolide for 24 h (* *P* < 0.001). Inhibitory effects on cell proliferation were assessed by MTT assay as described in the Materials and Methods. (**B**) The morphological change of TSGH cells upon sinulariolide treatments. TSGH cells were treated with dimethyl sulfoxide (control) or sinulariolide at the final concentrations of 5 μM, 10 μM and 15 μM, respectively, followed by observation the morphology of cells under the inverted light microscopy. (**C**) Cell migration assay showed that sinulariolide from 10 μM to 25 μM dose dependently suppresses TSGH cell migration (^#^ *P* < 0.05; * *P* < 0.001) and (**D**) Migrated TSGH cells were clearly reduced (15 μM of sinulariolide treated) compared with control cells at the 100× magnification vision. Scale bars (B, D) = 20 μM.

The anti-proliferative activity of sinulariolide was evaluated *in vitro* by the MTT assay. The MTT assay showed a significant anti-proliferative activity of sinulariolide on TSGH cells. As shown in Figure 2A, this effect was in a dose-dependent manner (15–30 μM). Moreover, the cell morphology was investigated and compared between control and sinulariolide-treated cells using the inverted light microscopy. The population of TSGH cells was reduced upon the treatment of sinulariolide at the concentration of 15 μM (Figure 2B). The result of the cell migration assay showed that sinulariolide exhibits the suppression of cell migration in a dose-dependent manner as the suppression rates were approximately 24%, 62% and 71%, at the concentration of 10, 15 and 25 μM, respectively (Figure 2C,D).

### 2.2. Sinulariolide-Treated TSGH Cells Adopt Apoptosis Characteristics

**Figure 3:**
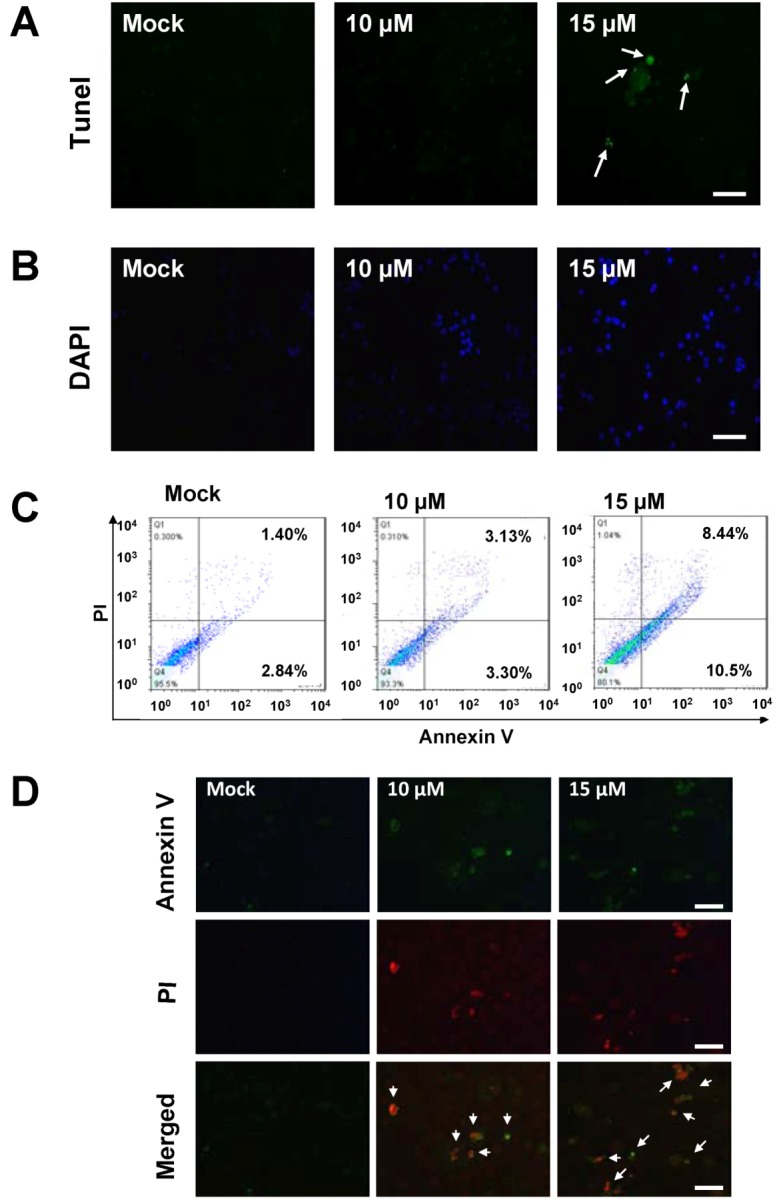
The appearance of apoptosis characteristics in the sinulariolide-treated TSGH cells. (**A**) Detection of apoptotic cells by TUNEL staining assay. TSGH cells were treated with DMSO or sinulariolide at the final concentrations of 10 μM and 15 μM for 24 h. The cells were harvested for TUNEL staining as described in the Materials and Methods section. Scale bar = 50 μM. (**B**) TSGH cells were treated with DMSO or sinulariolide at the final concentrations of 10 μM and 15 μM for 24 h. After harvest, the cells were then fixed with paraformaldehyde for immunofluorescent staining. TSGH cells were then stained with DAPI and visualized by fluorescent microscope. Scale bar = 50 μM. (**C**) Detection of externalization of phosphatidylserine (PS) from cell membrane after sinulariolide treatment stained by Annexin V-fluoresceinisothiocyanate (FITC)/propidium iodide (PI) analysis. Early apoptotic cells were increased after exposure to 10 μM and 15 μM sinulariolide and 15 μM sinulariolide elevated late apoptosis in TSGH cells. (**D**) Annexin V-FITC and PI analyze apoptotic TSGH cells upon sinulariolide treatment. The TSGH cells were stained by PI (red) as well as Annexin-V (green) after sinulariolide (at the final concentration of 10 μM and 15 μM) treatments. Scale bar = 50 μM.

It is now well established that some anti-cancer agents induce cell apoptosis [31]. In order to obtain more rigorous evidences of sinulariolide-induced cytotoxic effects in the TSGH cells through the apoptosis pathway, the tunnel stained assay was performed to evaluate the nuclear apoptosis caused by the treatment of sinulariolide. As a result, only sinulariolide-treated cells show a positive stain in the Terminal transferase dUTP Nick End Labeling (TUNEL) assay (Figure 3A). Second, the mock- and sinulariolide-treated cells were stained with 4’,6-diamidino-2-phenyl iodide (DAPI) and then examined for the morphological features of apoptosis. The results clearly show that sinulariolide induced when highly condensed at the concentration of 10 μM, and some massive apoptotic bodies were observed in the TSGH cells treated with 15 μM of sinulariolide (Figure 3B). 

To investigate whether the anti-cancer effect of sinulariolide is by means of induction of cell apoptosis, mock and sinulariolide-treated cells were stained with fluorescein isothiocyanatc-labeled Annexin-V and dye exclusion of propidium iodide (PI) for apoptosis flow cytometric detection on early apoptotic cells analyses. An apoptosis rate of 10.5% was observed in the sinulariolide-treated cells (at the concentration of 15 μM). By contrast, only 2.84% of apoptosis rate was observed for the untreated cells (lower right of Figure 3C), indicating that the sinulariolide induces the early apoptotic event while applied to the TSGH cells. Moreover, the late apoptosis cell rate was estimated as high as 8.44% on the sinulariolide-treated (15 μM) TSGH cells, whereas only 1.40% of late apoptosis rate was measured for the mock-treated cells (upper right of Figure 3C), suggesting that sinulariolide induces apoptosis on TSGH cells. 

To further validate the sinulariolide-induced apoptosis on the TSGH cells, mock- and sinulariolide-treated cells were co-stained with PI (red) and Annexin-V (green), followed by observing the positive cells using a fluorescent microscope. As shown in Figure 3D, some cells showed the positive staining for both PI and Annexin-V upon treatment with sinulariolide at the concentration of 10 μM and 15 μM. On the other hand, there was also no positive stain for PI, nor for Annexin V in the mock-treated cells. Altogether, these results clearly indicated that treatment with sinulariolide significantly induced the apoptosis of TSGH cells, as determinated by microscopic fluorescent observation and flow cytometric analysis, respectively.

### 2.3. Treatment of Sinulariolide Causes the Mitochondrial Depolarization in TSGH Cells

**Figure 4:**
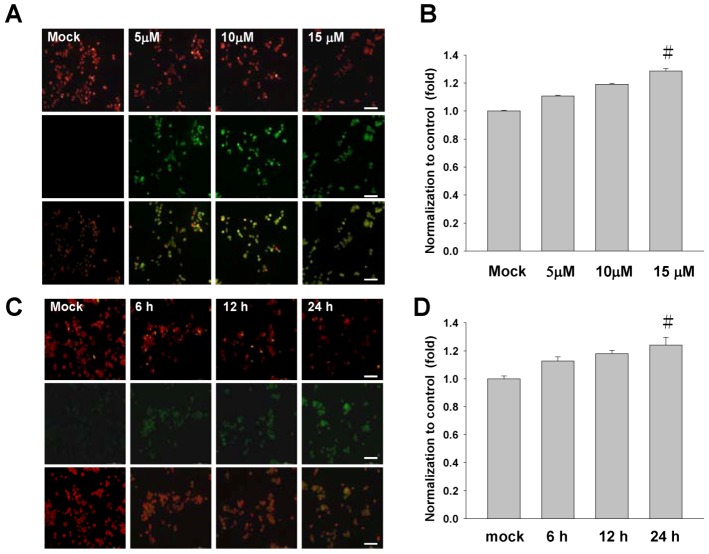
Sinulariolide induces the mitochondrial membrane depolarization in the TSGH cells. (**A**) Cells were treated as indicated, stained with JC-1 dye, incubated with cells for 20 min at 37 °C, 5% CO_2_ and imaged under fluorescence microscope at the emission wavelength of 580 nm (red, upper panels) and 530 nm (green, lower panels). (**B**) Percentage of green fluorescent cells from three independent experiments were quantified and presented as mean ± SD. ^#^ *P* < 0.05, as compared to control groups. (**C**) The time-course experiment of sinulariolide-treated TSGH cells at the final concentration of 15 μM for different time points as indicated, followed by staining with JC-1 dye and imaging as described above. (**D**) Percentage of green fluorescent cells were quantified and presented as described above. ^#^ *P* < 0.05. Scale bars (A, C) = 50 μM.

There is growing interest for the cell biology to evaluate changes in the mitochondrial membrane potential so as to define the role in initiating apoptosis and cell cycle. In this study, the mitochondrial membrane potential (∆Ψm) was assessed with JC-1 dye. The sinulariolide-treated TSGH cells showed a significant reduction in the red fluorescence while increased signals in the green fluorescence, suggest its loss of mitochondrial membrane potential due to the treatment of sinulariolide (Figure 4A,B). In addition, the time-course experiment was conducted to describe the early event of sinulariolide-induced mitochondrial depolarization. As shown in Figure 4C, the increase of positive stained of sinulariolide-treated cells were observed upon treatment, indicating that sinulariolide caused the cell apoptosis through the mitochondrial-related event (Figure 4C,D).

### 2.4. Sinulariolide Activates the Caspase-Dependent Pathway Resulting in Cell Apoptosis

**Figure 5:**
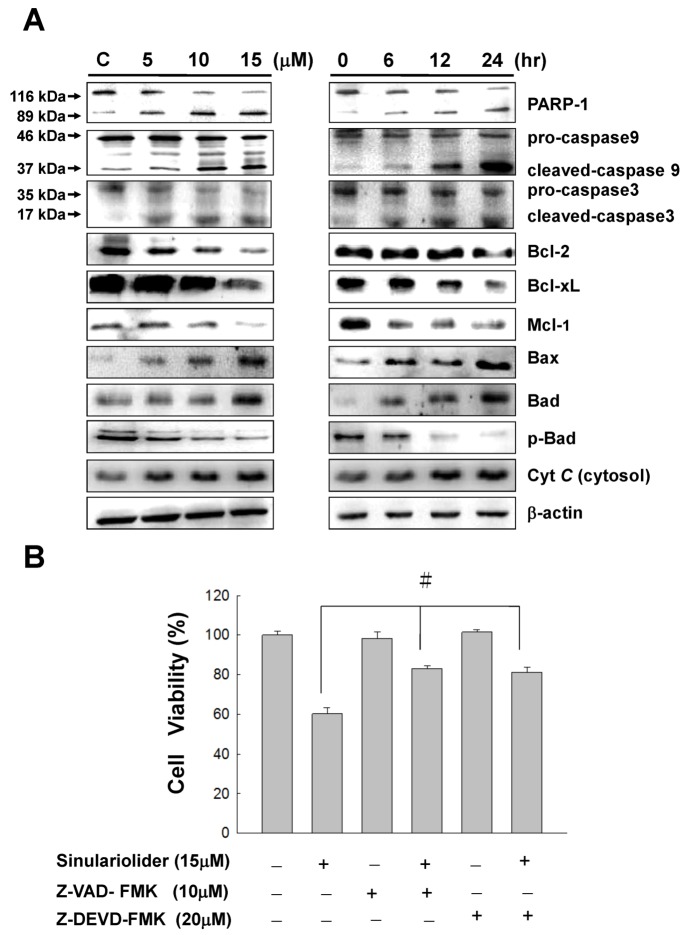
Sinulariolide induced apoptosis through the mitochondrial-related apoptosis pathway. (**A**) TSGH cells were treated with sinulariolide (0, 5, 10, and 15 μM) for 24 h and at the concentration of 15 μM for 6, 12, and 24 h, respectively. The detection of mitochondrial related apoptosis proteins were performed by western blotting analysis using specific antibodies as indicated. The results showed the changes of PARP-1, caspase-3, caspase-9, cleaved-caspase-3, cleaved-caspase-9, Cytochrome *C*, Bax, Bcl-2, Bcl-xl, Mcl-1, Bad and p-Bad expression in TSGH treated with sinulariolide. β-Actin was used for the protein loading control. (**B**) Two cell-permeant pan caspase inhibitors, Z-VAD-FMK and Z-DEVD-FMK, were added simultaneously with the treatment of sinulariolide in TSGH cells. The cells were then harvested at 24 h and subjected to MTT assay for the evaluation of cell viability. The related cell viabilities were determined from three independent experiments and quantified and presented as mean ± SD. ^#^ *P* < 0.05, as compared to control groups.

To further explore the potential mechanism of sinulariolide-induced apoptosis on the TSGH cells, several mitochondrial-related apoptosis pathway genes, including caspase-3, caspase-9, PARP-1, cytochrome *C*, Bax, Bcl-xl, Mcl-1, Bcl-2, Bad, and p-Bad respectively, were investigated by western blot analysis. The results were shown in Figure 5. The expression level of Bax, Bad and cytochrome *C* were increased in a time- and dose-dependent manner upon sinulariolide treatment. By contrast, the expression level of Bcl-2, Bcl-xl, Mcl-1 and p-Bad were decreased upon sinulariolide treatment. It has been well established that the mitochondrial cell death pathway is regulated by the Bcl-2 family members [32]. Especially the stoichiometry of Bax (pro-apoptotic member) and Bcl-2 (anti-apoptotic member) is critical for cytochrome *C* release and the following downstream caspase activation [33,34,35]. In this study, the expression level of Bax and cytochrome *C* were increased in the sinulariolide-treated cells, thus indicating that the sinulariolide-induced apoptosis associated with the activation of mitochondrial-related pathway on the TSGH cells.

To gain a comprehensive study on the sinulariolide-induced cell apoptosis. Caspase, another kinase protein which was reported as playing an important role in the regulation of cell survival, was examined, in addition to three mitochondrial-related apoptosis proteins which were described as the above [17]. The activation of both caspase-3 and caspase-9 are believed to encourage mitochondrial cell death signals [36].

The differential expression level of caspase-3 and caspase-9 were also reported as being involved in the cell apoptosis pathway. We next examined the changes of expression level of caspase-3 and caspase-9 upon sinulariolide treatment in the TSGH cells. Western blotting data showed that both pro-caspase-3 and pro-caspase-9 were downregulation in the sinulariolide treated cells. On the other hand, the expression level of cleaved caspase-3 and cleaved caspase-9 were elevated after sinulariolide treatment (Figure 5A). Similarly, the spliced form of PARP-1 (with the molecular size of 89 kDa, Figure 5A) was elevated upon sinulariolide treatment. This observation is consistent with the report showing that PARP-1 is cleaved by caspase during apoptosis [37]. 

Furthermore, two cell-permeant pan caspase inhibitors, Z-VAD-FMK and Z-DEVD-FMK, were added simultaneously with the treatment of sinulariolide in TSGH cells. As a result, both of the caspase inhibitors can inhibit the sinulariolide-induced cell apoptosis (Figure 5B). Taken together, these results show that the mitochondrial-related apoptosis pathway was activated upon sinulariolide treatments.

### 2.5. Sinulariolide-Induced the Activation of p38MAPK-ATF2 Pathway

**Figure 6:**
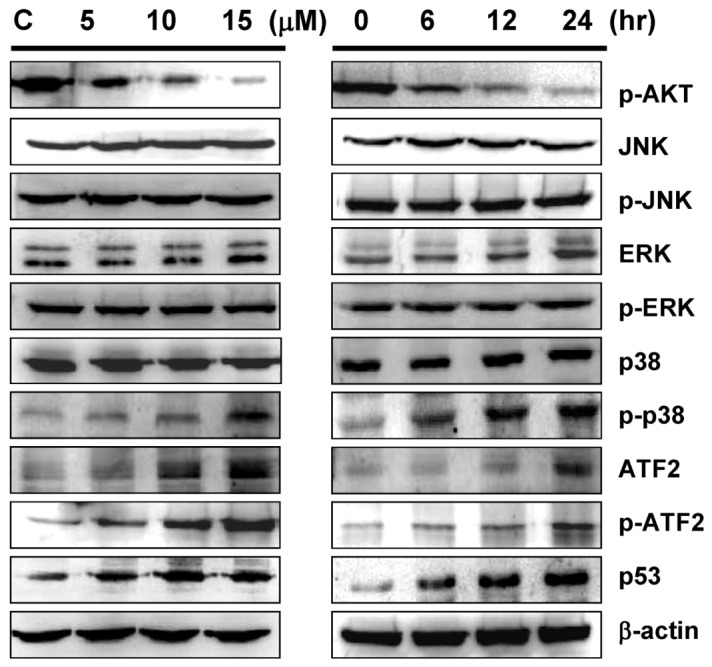
Western blot analyses of p38MAPK pathway related proteins upon sinulariolide treatment. TSGH cells were treated with sinulariolide (0, 5, 10, and 15 μM) for 24 h and at the concentration of 15 μM for 6, 12, and 24 h, as described in the Figure 5. The cell lysates were subsequently analyzed for the differential expression level of genes as indicated.

Mitogen-activated protein kinases (MAPKs) signal cascades which are found in all eukaryotes, have been demonstrated to play a central role in diverse biological processes, such as cell proliferation and apoptosis [38,39]. Here we examined the proteins, which are involved in the MAPKs pathway to elucidate the potential effects of sinulariolide on MAPKs activation in the apoptosis of TSGH cells. As shown by Western blot analysis, the expression of non-phosphorylated ERK, JNK and p38MAPK were not changed upon sinulariolide treatment, neither for the treatment of 15 μM of sinulariolide at various time points (Figure 6). By contrast, the accumulation of phosphorylated p38MAPK was significantly activated in a time- and dose-dependent manner, whereas no significant change of phosphorylation of JNK and ERK were observed (Figure 6). Additionally, we examined the expression level of ATF-2 and p53, which are the downstream proteins of p38MAPK pathway. The expression of ATF-2, phosphorylated p-ATF2 and p53 were upregulated in the sinulariolide-treated TSGH cells, suggesting that these proteins are also involved in this stress-activated signal pathway. Moreover, the upregulated expression level of p-Akt was also observed in the sinulariolide-treated cells. Since the phosphorylation of Bad by Akt could block anti-apoptotic activity in order to promote cell survival, the decreased expression of p-Akt indicating that sinulariolide caused cell apoptosis.

### 2.6. Inhibition of p38MAPK Activity Rescued the Cell Cytotoxicity of TSGH Cells by Sinulariolide

**Figure 7:**
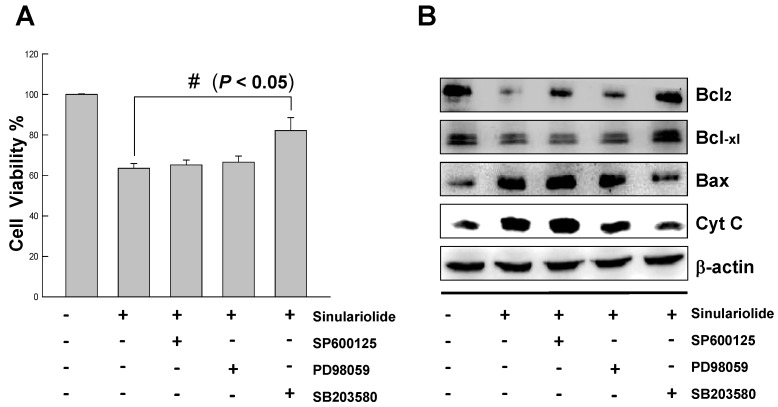
Inhibition of p38MAPK activity rescued the cell cytotoxicity of TSGH cells by sinulariolide.

To further characterize the sinulariolide-induced apoptosis is through the p38MAPK pathway, three different inhibitors were tested for the elucidation of p38MAPK-activated cell apoptosis upon sinulariolide treatment. As shown in Figure 7A, the increasing cell viability was observed from 60% to 80% upon treatment with SB203580 at the concentration of 15 μM in the sinulariolide-treated TSGH cells. By contrast, neither the PD98059 (the JNK-specific inhibitor) nor SP600125 (the MEK-specific inhibitor) were restored their cell viability due to the sinulariolide-induced apoptosis. Since the SB203580 is a p38MAPK-specific inhibitor, we conclude that sinuarliolide-induced cell apoptosis in the TSGH cells is through the p38MAPK signaling pathway. Moreover, the expression levels of Bax and cytochrome *C* were not increased in the sinulariolide-treated cells when the cells were pretreated with SB203580 (Figure 7B). Altogether, these results indicated that the p38MAPK pathway was partially involved in the sinulariolide-induced apoptosis on the TSGH cells.

## 3. Discussion

### 3.1. Sinulariolide Induces Apoptosis on the TSGH Cells

The anti-cancer effect of sinulariolide is due to its possessing the cytotoxic effects and apoptosis on the TSGH cells. In this study, we used the MTT assay, flow cytometer, and cell migration assay to demonstrate the sinulariolide induced the cytotoxic effect and cell apoptosis (Figure 2). The apoptotic morphological characters of TSGH cells induced by sinulariolide were also observed by flow cytometer as it showed that both early and late apoptosis occur upon sinulariolide treatments (Figure 3). Apoptosis plays important roles in removing DNA-damaged cells and maintaining tissue homeostasis [40]. The carcinogenesis progression of various cancers are primarily due to deregulation of apoptotic signal pathways [41]. Similarly, there were three other bladder cancer cell lines (RT4, T24 and BFTC905, respectively) have been chosen for the treatment of sinulariolide at the concentration of 15 μM and the MTT assay results showed that sinulariolide-treated cells also induced cell apoptosis (data not shown). Therefore, novel apoptotic inducers indeed provide effective and promising therapies against human cancers.

### 3.2. Sinulariolide Induces Apoptosis and Causes the Mitochondria Dysfunction on TSGH Cells

Activation of caspase cascade appears to be directly responsible for many agents to induce apoptosis in several different types of cancer cells [42]. It is also well known that the caspases are proteolytically activated during apoptosis. Upon receiving the proapoptotic signals, the activation of caspase 3 requires the activation of initiator caspases, such as caspase 8 or caspase 9 [42]. In this study, TSGH cells treated with sinulariolide increase the activation of caspase-3 and caspase-9 (Figure 5). 

We then conducted a mechanistic study to investigate whether the treatment of sinulariolide indeed activate apoptosis signals within the TSGH cells. Mitochondria-dependent apoptotic pathways can converge to mitochondria to promote the release of cytochrome *C*, along with other apoptotic factors, which can activate downstream targets to trigger apoptotic cell death [43]. The mitochondria-dependent apoptotic pathway has also been regulated by the Bcl-2 family protein. In particular, the activation of the pro-apoptotic Bax and Bad, and suppression of the anti-apoptotic proteins like Bcl-2, Bcl-xl, and Mcl-1 have been reported to regulate cytochrome *C* release from mitochondria [35,44].

Disruption of mitochondrial inter-membrane space proteins is believed to be the result from the remarkable event of mitochondrial outer membrane permeabilization, which are regulated by proteins of Bcl-2 family [45,46,47,48,49]. In the normal condition, Bcl-2 protein is usually localized on the mitochondrial outer membrane and serving as the central regulators of mitochondrial integrity. Without any stress, the Bcl-2 forms heterodimers with Bax (pro-apoptotic element), on the other hand, when the cells suffered stress, the Bax then translocated to the site of outer membrane, followed by inserting into the mitochondrial outer membrane. The insertion of Bax protein into the mitochondrial outer membrane led to increased membrane permeability and then leading to cytochrome *C* release by forming pores on the outer mitochondrial membrane [48]. 

Furthermore, cytochrome *C*, together with apoptosis protease-activating factor-1 (Apaf-1) and procaspase-9 forms [49], subsequently activates caspase-3, cleaving several caspase substrates leading to apoptosis [50]. Meanwhile, the protein caspase-3 has been demonstrated to cleave its substrate: PARP-1, inducing characteristic apoptotic progress, such as chromatin condensation and DNA chromatin fragmentation [17]. Besides, the cleaved-PARP-1 was also reported as playing an important role intriggering apoptosis, thus initiatiating apoptosis-inducing factor (AIF)-mediated cell death through a mechanism requiring Bax and calpains [51,52]. Here, we demonstrated that mitochondrial membrane potential in TSGH cells was attenuated within 24 h upon sinulariolide treatments (Figure 5). These results are rationally linked with the western blotting data of these mitochondrial apoptotic events such as enhancement of Bax expression, simultaneous inhibition of Bcl-2/Bcl-xL, subsequent Bax insert into mitochondria outer membrane, release cytochrome *C* and cleavage of caspase 3 and caspase 9 in sinulariolide-induced apoptosis in TSGH cells (Figure 5). In addition, the pro-apoptotic proteins such as Bax and Bad were upregulated; while the anti-apoptotic factors such as Bcl-2, Bcl-xl, and Mcl-1 were downregulated in the sinulariolide-treated TSGH cells (Figure 5). Taken together, these results imply that sinulariolide induced mitochondria-mediated apoptosis partially contributed to the cytotoxic effect of the TSGH cells. Interestingly, these findings are similar to the possible mechanisms of apoptosis induced by another soft coral extracted compound 11-dehydrosinulariolide in melanoma cell and 13-acetoxysarcocrassolide in bladder cancer cells in our previous study [27,53].

It is well known that MAPK signaling pathways are involved in mediating processes of cell growth, differentiation and apoptotic cell death. However, in our study, the expression level of ERK did not change in comparison with DMSO treated cells (Figure 6). In fact, many studies indicate that the ERK signaling pathway plays a crucial role in almost all cell functions and therefore requires exquisite control of its spatiotemporal activity. However, the ERK activity will mediate different anti-proliferative events, such as apoptosis, autophagy and senescence, depending on the cell type and stimulus. In some cells, ERK activity can promote either intrinsic or extrinsic apoptotic pathways by induction of mitochondrial cytochrome c release or caspase-8 activation, permanent cell cycle arrest or autophagic vacuolization. In this study, only the phosphorylated p38MAPK was significantly activated upon sinulariolide treatment, but not for the expression level of non-phosphorylated ERK, JNK, and p38MAPK, indicating that, at least, the ERK and JNK were not involved in this stress-activated signal pathway on TSGH cells. In other words, for the TSGH cells, ERK does not contribute to the stress-induced signal pathway.

JNK and p38MAPK pathways are activated in response to various stresses and chemicals [54]. Initiation of the p38MAPK pathway is required for induction of cellular apoptotic pathways in several different cellular models [55]. The stress-induced p38MAPK pathways activation was shown to regulate the cell cycle through modulation of p53 tumor suppressor protein and to reduce pro-survival Bcl-2 proteins expression [56,57]. Based on our observations, the expression level of JNK, ERK and p38MAPK did not change in comparison to untreated cells, and the phosphorylated ATF2 was increased upon sinulariolide treatment (Figure 6). The ATF2 has been described to be phosphorylated either by ERK, JNK or p38 [58]. Therefore, our results suggested that p38MAPK signaling, at least partially, is responsible for sinulariolide-induced apoptosis of TSGH cells.

## 4. Materials and Methods

### 4.1. Materials and Chemical Reagents

Rabbit anti-human antibodies against caspase-3, cleaved caspase-3, caspase-9, cleaved caspase-9, Bcl-2, Bcl-xl, Mcl-1, Bad, p38, p-p38, p-ERK, p-AKT and ATF2 and were from Cell Signaling Technology (Danvers, MA, USA). Rabbit anti-human antibodies against cytochrome C, p-Bad and p-ATF2 were from Epitomics (Burlingame, CA, USA). Rabbit anti-human JNK, p-JNK and ERK antibodies were obtained from ProteinTech Group (Chicago, IL, USA). Rabbit anti-human β-actin antibodies were obtained from Sigma (St Louis, MO, USA). Goat anti-rabbit and horseradish peroxidase conjugated IgG was from Millipore (Bellerica, MA, USA). 

Cell Extraction Buffer was obtained from BioSource International (Camarillo, CA, USA). Protease inhibitor cocktail, MTT, DMSO, JNK inhibitor SP600125, ERK inhibitor PD98059, and p38 inhibitor SB239063 were from Sigma (St Louis, MO, USA). PVDF (Polyvinylidene difluoride) membranes and Chemiliminescent HRP Substrate were from Pierce (Rockford, IL, USA). 

### 4.2. Cell Culture and the Treatment with Sinulariolide

Human urinary bladder carcinoma TSGH cells were obtained from the Bioresource Collection and Research Center (CBC, Food Industry Research and Development Institute, Hsin Chu, Taiwan). Cells were cultured as described in our previous study [30].

Sinulariolide was isolated from the soft coral *Sinularia flexibilis* was accomplished according to the reported procedures [24]. DMSO was used to dissolve sinulariolide. TSGH cells were treated with different concentrations of sinulariolide (5, 10, 15, 20, and 25 μM) and harvested after incubation for 24 h. All the experiments were repeated three times.

### 4.3. Cell Viability Assay

The cell cytotoxic effect of sinulariolide on TSGH cells were measured by MTT assay. TSGH cells were seeded 1 × 10^5^/cm^2^ in 96 well plates. After the addition of 5–25 μM sinulariolide for 24 h, the 50 μL MTT solution (1 mg/mL in phosphate buffered saline) was added to each well. The plates were then incubated at 37 °C for 4 h. DMSO (200 μL) was applied to each well to achieve solubility of purple-blue MTT formazan crystals. The optimal density (O.D) was measured at 595 nm by a microtiter ELISA reader with DMSO as the blank. 

### 4.4. Cell Migration Assay

The cell migration assay was performed according to the methods described by previously report [27]. TSGH cells in serum-free media were seeded onto polycarbonate membranes (8.0 μM, BD Biosciences, CA, USA) in the culture inserts. Cells treated with different concentrations (0, 5, 10, 15, and 20 μM) of sinulariolide and allowed to migrate for 24 h. Migrated cells were observed and counted under optics at 100× magnification.

### 4.5. Analysis Apoptosis by Flow Cytometry

The apoptosis induced by sinulariolide on TSGH cells were determined using Annexin V-FITC staining (Pharmingen, San Diego, CA, USA) according to manufacturer’s protocol on FACScan a flow cytometer (Becton-Dickinson, Mansfield, MA, USA) [59]. A total of 1 × 10^6^ cells were seed onto 5 cm petri dishes and treated with or without sinulariolide for 24 h and subsequently incubated with 10 μg/mL of annexin V-FITC and 20 μg/mL of PI at 37 °C for 30 min. Apoptotic distribution of sinulariolide-treated TSGH cells were analyzed by flow cytometry and Cell-Quest software (Becton-Dickinson, Mansfield, MA, USA).

### 4.6. Immunofluorescence Microscopy

TSGH cells (1 × 10^6^ cells) were pretreated with 0, 10 and 15 μM sinulariolide for 24 h. Then, the Annexin V-FITC Apoptosis staining reagent was added to the cells according to manufacturer’s protocol and incubated for 15 min at room temperature. Photomicrographs were obtained with a fluorescent microscope (Olympus IX71 CTS, Chinetek Scientific, Hong Kong, China). We used DeadEnd™ Fluorometric TUNEL System (Promega, Madison, WI, USA) to detect nuclear DNA fragmentation according to the manufacturer’s manual. The fragmented DNA of apoptotic cells by catalytically incorporating fluorescein-12-dUTP at 3′-OH DNA ends. The fluorescein-12-dUTP-labeled DNA can then be visualized directly by fluorescence microscopy. We also used a fluorescence microscope to identify the condense nuclei and fragmented that were stained with DAPI [14].

### 4.7. Mitochondrial Membrane Potential (ΔΨm) Assay Using Fluorescence Microscopy

Changes in the mitochondrial membrane potential (ΔΨm) after different treatments were examied by staining with the cationic dye JC-1 (5,5,6,6-tetrachloro-1,1,3,3-tetraethylbenzimidazolcarbocyanine iodide). JC-1 accumulates in the mitochondria of healthy cells and fluoresces red (560 nm). When the ΔΨm collapses, JC-1 uptake is limited to the cytoplasm where it fluoresces green (530 nm). TSGH cells were pretreated culture incubated with 10 mg/mL JC-1 (Biotium, Hayward, CA, USA). Cells were washed twice with PBS and observed by fluorescence microscopy (Olympus IX71 CTS, Chinetek Scientific, Hong Kong, China).

### 4.8. Protein Extraction and Protein Concentration Determination

TSGH cells treated with 0, 5, 10 and 15 μM sinulariolide for 24 h were lysed with Cell Extraction Buffer (BioSource International, Camarillo, CA, USA) and protease inhibitor cocktail (Sigma, St Louis, MO, USA). The protein concentration was determined according to the method describe by Bradford [60].

### 4.9. Mitochondria and Cytosol Fractionation

The separation of mitochondria and cytosol fraction is done using the Mitochondria/cytosol fractionation kit (BioVision, Miopitas, CA, USA) according to the manufacturer’s manual. Briefly, collect sinulariolide-treated and untreated TSGH cells at different time points as indicated. The cells were then centrifuged and washed with ice-cold PBS twice, followed by resuspending with 1× cytosol extraction buffer containing Dithiothreitol and Protease inhibitors. After incubation the cells on ice for 10 min, the cells were then homogenized in an ice-cold tissue grinder. The homogenates were then centrifuged and the supernatant is the cytosolic fraction, the pellet was then resuspended with mitochondrial extraction buffer.

### 4.10. Western Blotting Analysis

Twenty micrograms of each of the total cell lysates were separated by 12.5% SDS-PAGE and transferred to a PVDF membrane (Millipore) for 1.5 h at 400 mA using Transphor TE 62 (Hoefer). The PVDF membranes were then incubated with human PARP-1, caspase-3, cleaved-caspase-3, caspase-9, cleaved-caspase-9, Bax, Bcl-2, Bcl-xl, Mcl-1, Bad, p-Bad, cytochrome *C*, JNK, p-JNK, p38, p-p38, ERK, p-ERK, ATF2, p-ATF2, p53 and β-actin antibodies at 4 °C for 2 h or overnight. The membranes were washed three times in PBST (10 mM NaH_2_PO_4_, 130 mM NaCl, 0.05% Tween 20) and then probed with horseradish peroxidase conjugated antibodies (1:5000) for 1 h. The signals were visualized by using enhanced chemiluminesence Western Blotting Kits (Pierce).

### 4.11. Inhibitor Assessment

To further determine the effects of JNK, ERK, and p38MAPK on sinulariolide-induced proliferation arrest, a total of 1 × 10 cells per well in a 24-well plate were pre-incubated for 2 h with the following specific inhibitors, for JNK (SP600125), ERK (PD98059), and p38MAPK (SB239063) prior to sinulariolide administration. Afterwards, the cell proliferation rate was determined by the MTT assay.

## 5. Conclusion

**Figure 8:**
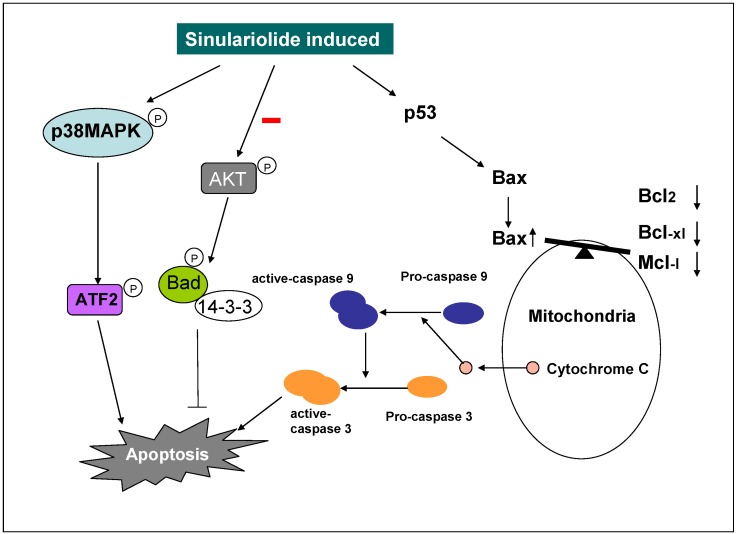
Illustration of sinulariolide induces cellular apoptosis through mitochondrial and p38MAPK-related pathway on TSGH cells.

This paper demonstrates the potent and selective cytotoxicity of sinulariolide towards TSGH cells. Sinulariolide exhibit the anti-cancer effect through the induction of cellular apoptosis. Our results indicated that the apoptosis induced by sinulariolide involves the activation mitochondrial-mediated apoptosis and p38MAPK pathway in TSGH cells (Figure 8). The differential expression levels of some apoptotic related proteins were investigated and the specific inhibitors were used to validate the potential mechanisms involved in the sinulariolide-induced apoptosis on the TSGH cells. Hence, further studies are needed to examine the *in vivo* efficacy and pharmacokinetic studies of sinulariolide.
